# Comparison of Breath and Blood Alcohol Elimination Rates of Non‐Alcohol Dependent People After a Heavy Drinking Session

**DOI:** 10.1111/acer.70437

**Published:** 2026-10-27

**Authors:** Kirsi Muuriaisniemi‐Skippari, Markus Keski‐Säntti, Teemu Gunnar, Kimmo Kuoppasalmi, Margareeta Häkkinen

**Affiliations:** ^1^ Forensic Toxicology Unit Finnish Institute for Health and Welfare Helsinki Finland; ^2^ Data Analytics and Reporting Unit Finnish Institute for Health and Welfare Helsinki Finland; ^3^ Department of Psychiatry University of Turku Turku Finland

**Keywords:** driving under the influence, ethanol, evidential breath analyzer

## Abstract

**Background:**

Data on elimination of blood alcohol concentration (BAC) and breath alcohol concentration (BrAC) are important for many purposes, such as in cases of driving under the influence (DUI). Current knowledge on BrAC elimination is based on studies with low‐alcohol concentrations, whereas in DUI cases alcohol concentrations are usually high. We studied BAC and BrAC elimination in real‐world drinking sessions with high alcohol concentrations.

**Methods:**

A total of 122 healthy volunteers consumed ethanol in seven study sessions of 11–13 h from October 2025 to February 2026, of which 43 females and 48 males gave samples during the confirmed ethanol elimination period. BAC was measured from whole‐blood samples. BrAC was analyzed with an evidential breath analyzer. We used linear regression models to assess background characteristics associated with BAC and BrAC elimination rates. We used bootstrapping to estimate the quantiles of the empirical elimination‐rate distributions and fitted quantile regression models to examine the robustness of the sample quantiles.

**Results:**

Mean and median BAC elimination rates for females were 0.200 and 0.201 g/kg/h, and for males 0.169 and 0.159 g/kg/h. Mean and median BrAC elimination rates for females were 0.096 and 0.095 mg/L/h, and for males 0.079 and 0.083 mg/L/h. Mean maximum BAC for females was 1.54 and for males 1.71 g/kg, and mean maximum BrAC was 0.73 and 0.78 mg/L, respectively. The 2.5% and 97.5% quantiles of BAC elimination rates for females were 0.116 and 0.292 g/kg/h, and of BrAC 0.034 and 0.139 mg/L/h, respectively. For males, these figures were 0.092 and 0.256 g/kg/h, and 0.028 and 0.137 mg/L/h.

**Conclusions:**

Estimated BAC and BrAC elimination rates were at or slightly above the upper range of previously reported values. For forensic applications, we suggest BrAC elimination rates of approximately 0.03 and 0.14 mg/L/h, corresponding to BAC elimination of 0.10 and 0.25 g/kg/h.

## Graphical abstract

We studied the elimination of blood alcohol concentration (BAC) and breath alcohol concentration (BrAC) in real‐world drinking sessions with high alcohol consumption. The mean and median elimination rates of females (BAC 0.200 and 0.201 g/kg/h, BrAC 0.096 and 0.095 mg/L/h) were higher than those of males (0.169 and 0.159 g/kg/h, 0.079 and 0.083 mg/L/h). Our results show that the variation in the elimination rates of BrAC was larger than that of BAC.

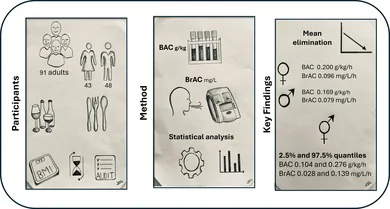

## 1 Introduction

Elimination of ethanol from the body is a key determinant of blood alcohol concentration (BAC) over time and fundamental to understanding alcohol pharmacokinetics. Knowledge of BAC elimination is important in clinical, research, and forensic settings, including the interpretation of alcohol exposure and cases of driving under the influence (DUI). The kinetics of BAC are well established, and the corresponding elimination rates have long been used in forensic interpretations and retrograde calculations (American Academy of Forensic Science (AAFS) 2024; Jones 2010). With advances in technology, increasing attention has been directed toward non‐invasive measurement methods, such as determination of breath alcohol concentration (BrAC). Evidential breath analyzers provide reliable on‐site testing of BrAC (Jones 2022; Jones and Cowan 2020). Despite their routine application in both civil and criminal calculations and interpretations, however, evidence on BrAC elimination remains limited compared with the extensive knowledge on BAC elimination.

The BAC elimination rate varies physiologically from 0.10 to 0.35 g/kg/h (Jones 2010). The lowest rates, 0.10–0.15 g/kg/h, apply to fasted subjects, whereas in non‐fasted subjects, the blood ethanol elimination rate is in the range of 0.15–0.25 g/kg/h (Jones 2010). In cases of moderate drinking, 0.15 g/kg/h is an average value, and for forensic purposes, the recommended value is 0.10–0.25 g/kg/h (American Academy of Forensic Science (AAFS) 2024). In cases of heavy alcohol drinking, the blood ethanol elimination rate might be as high as 0.25–0.35 g/kg/h (Jones 2010), or for some individuals, even higher (Jones 2008).

Elimination rates of BrAC are based on smaller studies consisting of 18–177 subjects (Dettling et al. 2009; Jachau et al. 2004; Jones and Andersson 2003; Pavlic et al. 2007; Reiter et al. 2020). In studies investigating both blood and breath alcohol elimination, estimated mean elimination rates of alcohol in the blood and breath, respectively, of 0.157 g/kg/h and 0.0805 mg/L/h (Jones and Andersson 2003), and 0.172 g/kg/h and 0.079 mg/L/h (Jachau et al. 2004) have been reported. Mean BAC and BrAC elimination rates of 0.187 g/kg/h and 0.092 mg/L/h for females, and 0.169 g/kg/h and 0.080 mg/L/h for males have also been reported (Dettling et al. 2009). For females, breath alcohol elimination rates have been proven to be higher than for males. Estimated mean breath alcohol elimination rates for females of 0.0865 mg/L/h (Jones and Andersson 2003), 0.087 mg/L/h (Pavlic et al. 2007), and 0.074 mg/L/h (Reiter et al. 2020) have been reported. For males, the corresponding estimations were 0.075 mg/L/h (Jones and Andersson 2003), 0.078 mg/L/h (Pavlic et al. 2007), and 0.065 mg/L/h (Reiter et al. 2020).

Elimination rates have so far been studied with rather low‐alcohol amounts and concentrations: 36 g of ethanol per person (Reiter et al. 2020), 0.5–1 g of ethanol per kg of body weight (Jachau et al. 2004), average alcohol consumption of 1.07 ± 0.23 g/kg body weight (Pavlic et al. 2007), and 0.40–0.65 g ethanol per kg of body weight (Jones and Andersson 2003). Maximum mean blood alcohol concentrations have been 0.9 ± 0.2 g/kg (Dettling et al. 2009). So far, no evidence has been gathered on whether BrAC elimination differs from BAC with higher alcohol concentrations. In real‐world DUI cases, alcohol concentrations are usually high. In Nordic countries, the median BAC among people arrested for drunken driving is 1.5–1.8 g/kg (Jones 2022). In Finland, the mean BAC in suspected DUI and driving under the influence of drugs (DUID) cases with a positive BAC finding during 2020–2025 was 1.59 g/kg (unpublished data from the National Bureau of Investigation). In fatal crashes involving impaired at‐fault drivers in Finland from 2019–2022, the mean BAC was 1.56 g/kg (Räty et al. 2026).

Our aim was to model breath alcohol elimination estimates for males and females based on various ethanol concentrations, including large concentrations. To investigate the likely lowest and highest elimination rates for breath alcohol concentration, we modeled the lowest and highest 2.5% and 5.0% quantiles.

## 2 Materials and Methods

### 2.1 Subjects and Conditions

A total of 122 healthy volunteers (men and women aged 19–69 years) participated in one of the seven study sessions, each lasting approximately 11–13 h, conducted in October 2025, December 2025, or February 2026. Participants with varying body compositions and drinking histories were recruited, with the aim of forming a study population broadly comparable to the Finnish population of licensed drivers.

Exclusion criteria included inability or unwillingness to provide a breath sample using an evidential breath alcohol analyzer, a baseline breath alcohol concentration other than zero, use of centrally acting medication (CNS), narcotics, or other intoxicating substances (such as opioids or benzodiazepines) within the preceding week, a diagnosed substance use disorder, pregnancy or breastfeeding, fever, severe pulmonary disease, and any condition in which alcohol consumption is considered an acute health risk.

Participants were recruited by email through the forensic toxicology unit of the Finnish Institute for Health and Welfare (THL), the police, and the Finnish Border Guard. To improve the representativeness with regard to the general population, people receiving recruitment emails were allowed to recruit acquaintances to participate in the study. At the beginning of the study session, all participants gave written informed consent. Participation was anonymous, and each participant received a unique study identifier not linkable to personal data.

The participants consumed alcohol at a steady rate for an average duration of 6–7 h under medical supervision and chose the type and amount consumed themselves. Available beverages included beer and other low‐alcohol drinks, wine, spirits, and non‐alcoholic alternatives. Study personnel recorded the amount of alcohol consumed, beverage type, and time of consumption. Meals and snacks were provided throughout the study sessions. Blood samples and evidential breath alcohol measurements were collected at 1–2‐h intervals during the absorption period and approximately every hour during the elimination period, with the order of blood and breath sampling randomized in each sampling round. Breath alcohol measurements were performed no earlier than 15 min after alcohol consumption to avoid residual mouth alcohol effects (Gullberg 1992; Jones 2022; Lindberg et al. 2015). Samples were collected across different phases of intoxication. We aimed to collect at least three samples during the alcohol absorption period (6–7 h) and four during the elimination period (3–5 h). The maximum number of sampling rounds per participant was seven. To ensure that four samples could be collected during the elimination phase, alcohol consumption ceased 3–5 h before the end of the study session.

### 2.2 Blood Sampling and Blood Alcohol Concentration Analysis

Venous blood and breath samples were collected from each participant in randomized order. Venous blood was drawn from the antecubital vein into gray‐stoppered evacuated tubes containing sodium fluoride and potassium oxalate (Vacuette, Item No. 454297, Greiner Bio One, Kremsmüster, Austria). The puncture site was cleaned with alcohol‐free wipes (Reliwipe Alcohol Free Cleansing Wipe, REF:741, Reliance Medical Ltd., Staffordshire, England) or sterile water. Samples were mixed by inversion and stored at 4°C until analysis.

BAC was determined by headspace gas chromatography with flame ionization detection (HS‐GC‐FID) using two PerkinElmer Clarus 590 instruments equipped with Turbomatrix 110 HS autosamplers (PerkinElmer, Shelton, CT, USA). Separation was achieved on dual columns (Rtx BAC 1 and Rtx BAC 2 Restek, Bellefonte, PA, USA) in both instruments. HS and GC operating conditions followed a validated forensic protocol.

Prior to analysis, blood samples (100 μL), calibrators (6 points), blanks, and controls were diluted sixfold using either n‐propanol or 2‐butanol (0.20 g/kg) as internal standards. When converted to the unit g/100 mL, 1 g/kg (‰) corresponds to 0.1055 g/100 mL. Dilutions were performed using a Hamilton Microlab 600 (Hamilton Co., Reno, NV, USA), after which samples were transferred to sealed 20‐mL HS vials.

Two aliquots of each blood sample were analyzed using both instruments and both columns, yielding four BAC results per sample; the mean value was used for statistical analyses. Acceptance criteria required agreement between parallel measurements of ≤ 0.035 g/kg at BAC ≤ 0.500 g/kg, or ≤ 7% at higher concentrations. Method performance characteristics included a limit of quantification of 0.10 g/kg and linearity from 0.10 to 6.0 g/kg.

The quantitative analytical method for ethanol determination in blood samples is routinely used at the national level for the analysis of blood alcohol concentrations in DUI cases. The testing method is fully validated, accredited by the Finnish Accreditation Service (FINAS) in accordance with EN ISO/IEC 17025, and its performance is continuously monitored through participation in proficiency testing (PT) rounds (interlaboratory comparisons), which provide independent verification of analytical accuracy.

### 2.3 Breath Alcohol Analysis

The Intoxilyzer 9000 (CMI Inc., Owensboro, KY, USA) is a quantitative infrared evidential breath alcohol analyzer used by the Finnish Police and Border Guard authorities for DUI casework. We used ten instruments for the study; three to six devices were operated simultaneously in the same room. Each instrument was connected to an external dry ethanol gas standard Calgaz 6DM cylinder (Calgaz HQ, Houston, TX, USA), and performance was verified by an initial calibration check. The nominal concentration of the external standard was 0.381 mg/L (acceptable range 0.362–0.400 mg/L).

BrAC was measured using infrared absorption at four wavelengths. Participants provided breath samples in random order according to a predefined schedule, as close as possible to blood sampling. Each subject performed a prolonged deep exhalation into a heated inlet tube. Valid samples required a minimum exhalation time of 5 s, volume of 1.5 L, and flow rate of 0.15 L/s, in compliance with the requirements of OIML R 126:2012 (E) (International Organization of Legal Metrology (OIML) 2012).

Duplicate BrAC measurements were obtained at 3‐min intervals. The instrument followed the standard operational cycle, including air blanks, diagnostics, mouthpiece tests, subject breath tests, and internal standard checks before and after subject testing. The target concentration of the internal standard was 0.22 mg/L (acceptable range 0.19–0.25 mg/L). The instrument reports 0.00 mg/L at concentrations ≤ 0.03 mg/L, which represents the limit of quantitation. Duplicate results were accepted if they differed by ≤ 0.04 mg/L and ≤ 12%. We used mean duplicate BrAC values (mg/L) for statistical analysis and compared them with the mean of four BAC results (g/kg).

### 2.4 Statistical Analysis

Statistical analyses focused on the alcohol elimination rates in the study population as measured by change in BAC (g/kg/h) and BrAC (mg/L/h). The lowest BAC value included in the statistical analysis was 0.187 g/kg. We classified a measurement as an elimination phase sample when the following criteria were met for the blood samples: (1) the measured BAC was lower than that measured in the preceding sample from the same participant, and (2) the sample was collected after the cessation of drinking. Furthermore, we defined an elimination phase interval as a sequence of two consecutive samples, both of which were classified as elimination phase samples. Based on the blood samples, we identified 139 elimination phase intervals from 91 participants. If a participant had multiple consecutive elimination phase intervals, we combined these intervals into a single elimination phase interval extending from the first elimination phase sample to the last elimination phase sample. The overall change was determined from the first and last elimination phase samples, irrespective of the number of intermediate measurements. We calculated the change in alcohol concentration in the original unit of measurement (g/kg for BAC and mg/L for BrAC) and divided it by the corresponding time interval to obtain elimination rates expressed as g/kg/h for BAC and mg/L/h for BrAC.

For the statistical analyses, linear regression models were first fitted to assess whether background characteristics were associated with BAC and BrAC elimination rates. Secondly, selected quantiles of the empirical elimination‐rate distribution and their confidence intervals were estimated using bootstrapping with 5000 resamples. Lastly, as our interest was in the tails of the outcome distribution, quantile regression models were fitted and bootstrapped as a sensitivity analysis to examine the robustness of the sample quantiles.

### 2.5 Ethics Statement

The Research Ethics Committee of the Hospital District of Helsinki and Uusimaa approved the study (approval number HUS/7562/2025). All participants gave written consent before entering the study.

## 3 Results

The 91 participants included in the main analysis contributed a total of 599 measurement occasions. The pairwise Pearson correlation coefficient between the BAC and BrAC measurements was *r* = 0.988, corresponding to *r* ^2^ = 0.976.

Table 1 shows the mean elimination rates and characteristics of the 91 subjects studied. Mean BAC and BrAC elimination rates for females were 0.20 g/kg/h and 0.096 mg/L/h, respectively, and for males 0.17 g/kg/h and 0.079 mg/L/h. The mean midpoint of the elimination phase was approximately 2 h after cessation of drinking. Mean alcohol intake during the study period was high: for females, 2.0 g per kg of body weight, and for males 2.3. Mean maximum BAC and BrAC were 1.5 g/kg and 0.73 mg/L for females, and for males 1.7 g/kg and 0.78 mg/L. The study subjects were mainly middle‐aged people with a mean BMI of 27.7. Of the females, 60% had an AUDIT‐C score ≥ 4, and 42% of the males had an AUDIT‐C score ≥ 6. Weekly or monthly heavy episodic drinking (≥ 6 units of alcohol on a single occasion) was reported by 12% of females and 46% of males.

**TABLE 1:** Mean (standard deviation) blood alcohol concentration (BAC) and breath alcohol concentration (BrAC) elimination rates and characteristics of the 91 subjects studied.

Characteristic	Females, *n* = 43	Males, *n* = 48	Total, *n* = 91
BAC elimination (g/kg/h)	0.200 (0.046)	0.169 (0.042)	0.184 (0.047)
BrAC elimination (mg/L/h)	0.096 (0.024)	0.079 (0.028)	0.087 (0.027)
Midpoint BAC elimination^a^ (min)	128.3 (46.3)	121.6 (29.6)	124.8 (38.3)
Midpoint BrAC elimination^a^ (min)	124.6 (44.1)	117.3 (27.0)	120.8 (36.1)
Age (years)	41.6 (9.7)	43.2 (11.6)	42.5 (10.7)
Height (cm)	167.0 (4.8)	179.4 (5.4)	173.5 (8.1)
Weight (kg)	76.6 (15.4)	89.5 (13.5)	83.4 (15.8)
BMI (kg/m^2^)	27.50 (5.70)	27.89 (4.57)	27.70 (5.11)
Waist (cm)	87.0 (14.6)	94.0 (11.8)	90.7 (13.6)
AUDIT score	4.5 (1.7)	7.0 (3.5)	5.8 (3.1)
AUDIT‐C score	3.8 (1.3)	5.2 (1.9)	4.5 (1.8)
*V*_d_ (L)	0.55 (0.05)	0.65 (0.04)	0.60 (0.07)
TBW (L)	36.6 (3.9)	47.9 (4.4)	41.6 (7.8)
Ingested alcohol (g)	146.6 (35.4)	206.2 (43.8)	178.0 (49.8)
Ingested alcohol (g/kg of body weight)	1.96 (0.48)	2.33 (0.51)	2.15 (0.52)
Max BAC (g/kg)	1.54 (0.53)	1.71 (0.51)	1.63 (0.53)
Max BrAC (mg/L)	0.73 (0.25)	0.78 (0.23)	0.76 (0.24)
Midpoint BAC^b^ (g/kg)	1.20 (0.55)	1.44 (0.53)	1.33 (0.55)
Midpoint BrAC^b^ (mg/L)	0.55 (0.26)	0.63 (0.23)	0.59 (0.25)

Abbreviations: AUDIT, Alcohol Use Disorder Identification Test; AUDIT‐C, first three questions of the AUDIT; BMI, body mass index; TBW, total body water based on the Watson et al. equation with height, weight, and age (Maskell et al. 2019); *V*_d_, volume of distribution.

^a^ Midpoint BAC/BrAC elimination denotes the time (in minutes) from cessation of the drinking to the midpoint of an elimination phase.

^b^ Midpoint BAC/BrAC are calculated as participant‐level (start value + end value)/2, estimated mean midpoint values.

Linear regression models estimating the elimination rate of BAC and BrAC showed limited explanatory power. In the base models including sex as the only explanatory variable, the adjusted R‐squared values for BAC and BrAC were 0.108 and 0.084, respectively. The sex difference in the elimination rates was statistically significant in the univariable model (*p* = 0.001). We then fitted univariable models for the predefined background characteristics, as well as multivariable models including sex and each characteristic, and in some cases, interaction terms. None of these models substantially improved the model fit, and the adjusted R‐squared values remained below 0.15. The tested models are listed in the Tables S1 and S2.

Median alcohol elimination values from blood and breath in the data for females were 0.201 g/kg/h and 0.095 mg/L/h, and for males 0.159 g/kg/h and 0.083 mg/L/h. Bootstrap resampling was used to estimate 2.5%, 5%, 50%, 95%, and 97.5% quantiles of the elimination rates and their confidence intervals; we present this in Figure 1. The lowest 2.5% quantile for BrAC elimination rate was 0.028 mg/L/h, and for BAC elimination rate 0.104 g/kg/h (Figure 1), rather close to the suggested lower limit for forensic calculations of 0.10 g/kg/h. At the higher end, the 97.5% quantile, at 0.139 mg/L/h or 0.276 g/kg/h, was somewhat higher than the suggested higher limit for forensic calculations of 0.25 g/kg/h.

**FIGURE 1:**
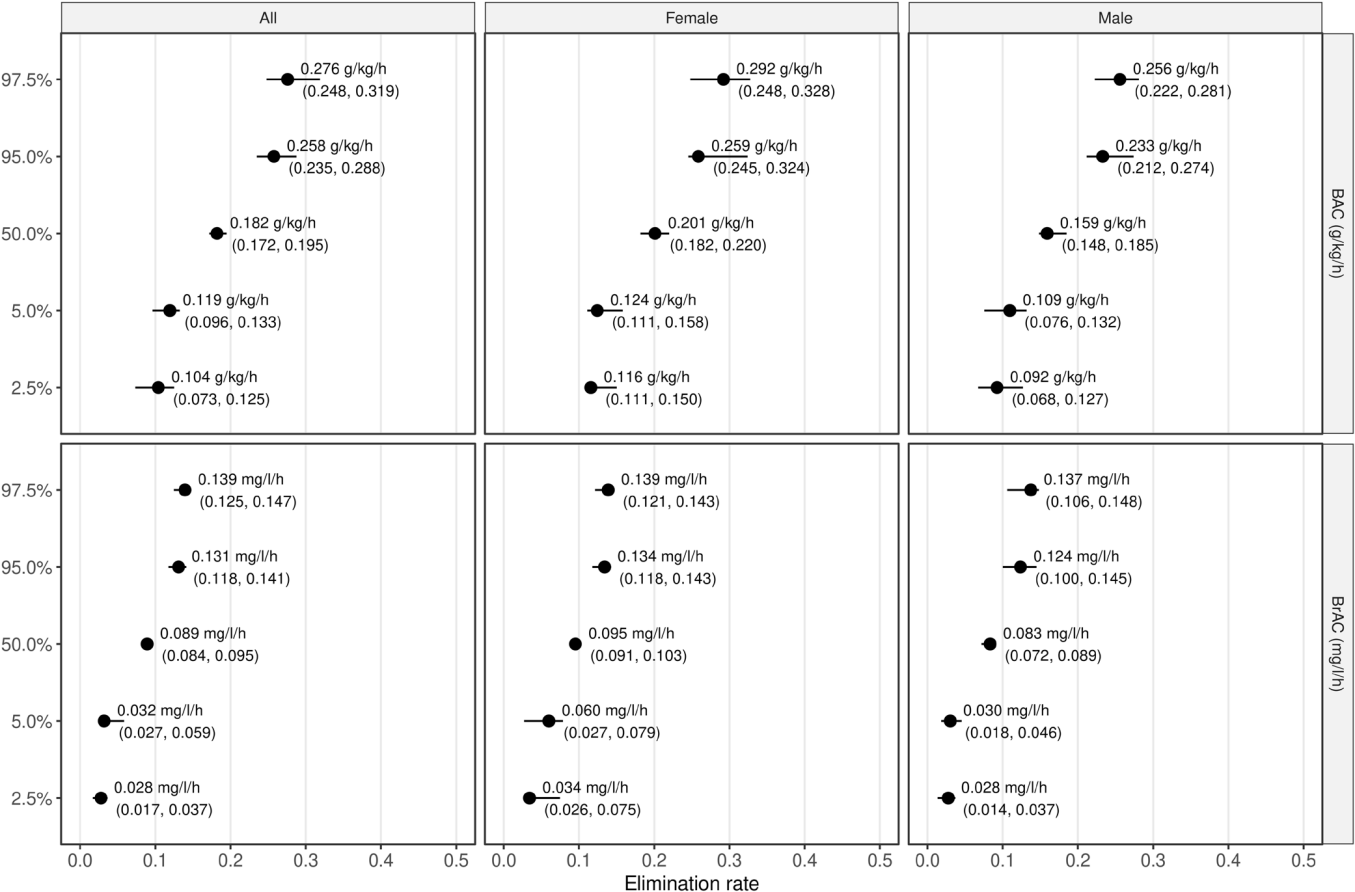
Quantiles of blood alcohol concentration (BAC) and breath alcohol concentration (BrAC) elimination rates based on bootstrapping of the unadjusted model. The solid line represents the 95% confidence intervals of the bootstrapped BAC and BrAC estimates.

Because high alcohol consumption has been associated with faster ethanol elimination rate (Jones 2008), we conducted a sensitivity analysis to assess whether the higher elimination rates observed at high ethanol concentrations could reflect alcohol consumption patterns of the study population rather than an effect of high ethanol concentrations per se. After excluding females with AUDIT scores ≥ 6 (*n* = 13) and males with AUDIT scores ≥ 8 (*n* = 15), no substantial differences were observed between females and males with respect to age, BMI, or volume of distribution (V_d_). Mean alcohol intake during the study period remained high, averaging 1.9 g/kg body weight in females and 2.2 g/kg body weight in males. The mean maximum BAC and BrAC values were 1.4 g/kg and 0.65 mg/L for females, and 1.5 g/kg and 0.70 mg/L for males. The mean elimination rates for BAC and BrAC for females were 0.200 g/kg/h and 0.094 mg/L/h, and for males, 0.167 g/kg/h and 0.078 mg/L/h. Thus, the results remained stable in the sensitivity analysis. Furthermore, neither AUDIT nor AUDIT‐C scores were significantly associated with the ethanol elimination rates in the linear regression models (Tables S1 and S2).

## 4 Discussion

We measured breath alcohol elimination rates after cessation of drinking among non‐alcohol‐dependent individuals after a heavy drinking session. Mean BrAC elimination rate for females was 0.096 mg/L/h, and for males 0.079 mg/L/h. These results are somewhat higher than previously reported mean results of 0.074–0.092 mg/L/h for females, and at the higher end of previously reported mean values of 0.065–0.080 mg/L/h for males (Dettling et al. 2009; Jachau et al. 2004; Jones and Andersson 2003; Pavlic et al. 2007; Reiter et al. 2020).

BrAC elimination rates among males were similar to another uncontrolled, real‐world study of BrAC elimination by Pavlic et al. (2007), whereas among females, our mean BrAC elimination‐rate estimates were somewhat higher, 0.096 vs. 0.087 mg/L/h. The characteristics of the study population and amount of alcohol consumed in the two studies were different; in our study, the subjects covered a broader age distribution and were on average older with higher BMI, which may partly explain the difference in BrAC elimination among females. In addition, in our study the time of alcohol consumption was longer, and mean consumption of alcohol was about two‐fold, which influences alcohol kinetics and hampers comparisons between these studies. When compared to controlled studies (Jachau et al. 2004; Jones 2010; Reiter et al. 2020), the BrAC elimination rates in our uncontrolled study were somewhat higher. This might be due to our real‐world study design, with no exact clinical control variables. In our uncontrolled study design, we could not control, for example, eating and stomach contents, which influence alcohol absorption. In real‐world cases, these generic external factors predict elimination rates poorly, even though they perform well in laboratory trials which control eating, drinking, type of alcoholic beverage, and stomach contents.

Sex, age, height, weight, and alcohol consumption habits typically contribute to BAC and its metabolism. The body structures of our study population resembled the general Finnish population according to the Healthy Finland Survey (Sääksjärvi et al. 2025). For example, the mean BMI among females in our study was 27.5 kg/m^2^ and among males 27.9 kg/m^2^, whereas among the general population these figures are 27.7 and 27.9 kg/m^2^ (Lehtoranta et al. 2023). Compared to the general population, risky alcohol consumption was more common in our study population. In the general Finnish population aged 20 and older, based on the AUDIT‐C questionnaire and a screening limit of 6 points for men and 4 points for women and men aged ≥ 65, a third of men and less than a fifth of women were classified as engaging in risky alcohol use (Mäkelä et al. 2023). In our study, these figures were much higher, 42% for males and 60% for females. In the general population, 29% of men and 10% of women consume ≥ 6 alcohol units on a single occasion monthly or more often, and among men aged 55–64 years, this figure is as high as 36% (Mäkelä et al. 2023). Even though 60% of the females in our study engaged in risky alcohol consumption, only 12% consumed ≥ 6 units monthly or more often, whereas this figure for males, 46%, was higher in our study than in the general population. Although high alcohol consumption has been associated with accelerated ethanol elimination (Jones 2008), our sensitivity analysis did not indicate a substantial effect of risky alcohol use on the elimination rates. Excluding participants with AUDIT scores indicative of risky alcohol use (females ≥ 6 and males ≥ 8; total *n* = 28) produced only minor changes in the BAC and BrAC elimination rate, and the overall findings remained consistent. Therefore, differences in risky alcohol consumption do not appear to fully explain the high elimination rates observed in our study.

A strength of our study is the large real‐world sample set covering a wide range of alcohol concentrations among females and males with different ages, body structures, and drinking habits. Our analysis of the quantiles is based on empirical data and, as such, does not assume normality of the elimination‐rate distribution. Alcohol consumption in the study sessions was higher than in previous BrAC elimination studies, resembling actual DUI cases. The mean highest BAC in our study, 1.63 g/kg, was close to the mean BAC in DUI cases, 1.59 g/kg (unpublished data from National Bureau of Investigation), and the mean BAC in fatal crashes involving impaired at‐fault drivers, 1.56 g/kg (Räty et al. 2026). In contrast to many other BAC and BrAC elimination studies, we measured BAC from whole‐blood samples, whereas other studies have measured BAC in serum utilizing serum/blood conversion factors (Dettling et al. 2009; Jachau et al. 2004; Pavlic et al. 2007). This might lead to differences in reported BAC values, as the use of serum‐to‐whole‐blood conversion factors (e.g., ≥ 1.2) accounts for variability in blood composition but may introduce systematic differences as well as additional inter‐individual variability (Jones and Tilson 2023), given that these factors represent average estimates rather than exact equivalence.

One limitation of our study is that, although the study sessions allowed people to drink large amounts of alcohol, alcohol dependency was an exclusion criterion, and therefore, these results may not be generalizable to DUI cases in which alcohol dependency is common, usually leading to faster alcohol metabolism and elimination rates (Jones 2008, 2010). Furthermore, due to the limited sample size, bootstrapped confidence intervals for the extreme ends of the distribution are based on a small number of observations and should therefore be interpreted with caution. Instead of fixed limits for alcohol calculations, Maskell et al. (2026) recommend using percentile ranges determined from an appropriate population using bootstrapping to estimate the relevant percentile ranges. For civil cases, they recommend the 25th and 75th percentiles (50% range), and for criminal cases the 0.5th and 99.5th (99% range) or 2.5th and 97.5th percentiles (95% range), depending on the amount of data available (Maskell et al. 2026). In our data, the sample size was insufficient for estimation of the 0.5th and 99.5th percentiles.

In conclusion, in our real‐world drinking study with high ethanol concentrations, the estimated BAC and BrAC elimination rates were at the upper end of previously reported values, with mean elimination rates slightly higher than previously reported values. For forensic purposes, a need exists for BrAC elimination rates comparable to BAC 0.1 and 0.25 g/kg/h. For BrAC, the estimated corresponding elimination range in our study was wider, reflecting larger variation in BrAC than BAC elimination rates. According to the bootstrapped 2.5% and 97.5% quantiles (95% range) in our data, the BrAC elimination‐rate range is approximately 0.03 to 0.14 mg/L/h.

## Funding

The National Police Board of Finland funded the study.

## Conflicts of Interest

The authors declare no conflicts of interest.

## Supporting information


**Table S1:** Linear regression models estimating the blood alcohol concentration (BAC) elimination rate.
**Table S2:** Linear regression models estimating the breath alcohol concentration (BrAC) elimination rate.

## Data Availability

The data are not publicly available due to privacy or ethical restrictions.
