# Supplementary material for: Comparison of Breath and Blood Alcohol Elimination Rates of Non‐Alcohol Dependent People After a Heavy Drinking Session
Source: Alcohol Clin Exp Res (Hoboken). 2026 Oct 27;50(11):e70437. doi: 10.1111/acer.70437 (PMC13711466; doi:10.1111/acer.70437)
Supplement: Supplementary file 1 [file ACER-50-0-s001.docx]

# Supplementary tables

Table S1. Linear regression models estimating the blood alcohol concentration (BAC) elimination rate. “:” denotes interaction.

| Model predictors | Adj R^2^ | R^2^ | df | AIC | BIC |
| --- | --- | --- | --- | --- | --- |
| Sex | 0.108 | 0.118 | 1 | -306.594 | -299.061 |
| Sex + age | 0.103 | 0.123 | 2 | -305.027 | -294.983 |
| Sex + weight | 0.099 | 0.119 | 2 | -304.622 | -294.579 |
| Sex + BMI | 0.101 | 0.121 | 2 | -304.827 | -294.784 |
| Sex + waist | 0.098 | 0.118 | 2 | -304.602 | -294.559 |
| Sex + height + weight | 0.134 | 0.163 | 3 | -307.360 | -294.806 |
| Height + weight | 0.141 | 0.160 | 2 | -309.043 | -298.999 |
| Sex + height | 0.144 | 0.163 | 2 | -309.360 | -299.316 |
| Sex + height + sex:height | 0.135 | 0.164 | 3 | -307.387 | -294.833 |
| Sex + TBW | 0.099 | 0.119 | 2 | -304.629 | -294.586 |
| Sex + V_d_ | 0.098 | 0.118 | 2 | -304.598 | -294.555 |
| TBW | 0.081 | 0.091 | 1 | -303.790 | -296.257 |
| V_d_ | 0.061 | 0.071 | 1 | -301.855 | -294.323 |
| Sex + NCS of midpoint BAC elimination^a^ | 0.143 | 0.190 | 5 | -306.339 | -288.763 |
| Sex + age + midpoint BAC elimination^a^ | 0.102 | 0.132 | 3 | -304.027 | -291.472 |
| Sex + height + weight + midpoint BAC elimination^a^ | 0.132 | 0.170 | 4 | -306.14 | -291.075 |
| Sex + total alcohol consumed | 0.100 | 0.120 | 2 | -304.793 | -294.749 |
| Sex + alcohol consumed per kg of body weight | 0.101 | 0.121 | 2 | -304.848 | -294.805 |
| Alcohol consumed per kg of body weight | 0.018 | 0.028 | 1 | -297.756 | -290.223 |
| Total alcohol consumed | 0.048 | 0.058 | 1 | -300.603 | -293.071 |
| AUDIT | -0.004 | 0.008 | 1 | -295.828 | -288.295 |
| Sex + AUDIT | 0.102 | 0.122 | 2 | -304.924 | -294.881 |

^a^Midpoint BAC elimination denotes the time (in minutes) from cessation of drinking to the midpoint of an elimination phase.

Adj R^2^: adjusted coefficient of determination, AIC: Akaike Information Criterion, AUDIT Alcohol Use Disorders Identification Test, BIC: Bayesian Information Criterion, df: degrees of freedom, BMI: body mass index, NCS: Natural Cubic Spline, V_d_: volume of distribution, TBW: total body water based on the Watson et al. equation with height, weight, and age (Maskell et al., 2019).

Table S2. Linear regression models estimating the breath alcohol concentration (BrAC) elimination rate. “:” denotes interaction.

| Model predictors | Adj R^2^ | R^2^ | df | AIC | BIC |
| --- | --- | --- | --- | --- | --- |
| Sex | 0.084 | 0.094 | 1 | -400.78 | -393.248 |
| Sex + age | 0.077 | 0.098 | 2 | -399.159 | -389.115 |
| Sex + weight | 0.074 | 0.094 | 2 | -398.830 | -388.787 |
| Sex + BMI | 0.081 | 0.102 | 2 | -399.568 | -389.525 |
| Sex + waist | 0.081 | 0.101 | 2 | -399.515 | -389.472 |
| Sex + height + weight | 0.110 | 0.139 | 3 | -401.469 | -388.914 |
| Height + weight | 0.118 | 0.138 | 2 | -403.290 | -393.246 |
| Sex + height | 0.118 | 0.138 | 2 | -403.297 | -393.254 |
| Sex + Height + sex:height | 0.114 | 0.144 | 3 | -401.912 | -389.357 |
| Sex + TBW | 0.073 | 0.094 | 2 | -398.788 | -388.745 |
| Sex + V_d_ | 0.076 | 0.096 | 2 | -399.014 | -388.970 |
| TBW | 0.054 | 0.065 | 1 | -397.917 | -390.384 |
| V_d_ | 0.060 | 0.070 | 1 | -398.436 | -390.903 |
| Sex + NCS of midpoint BrAC elimination^a^ | 0.113 | 0.162 | 5 | -399.899 | -382.323 |
| Sex + age + midpoint BrAC elimination^a^ | 0.079 | 0.110 | 3 | -398.396 | -385.842 |
| Sex + height + weight + midpoint BrAC elimination^a^ | 0.112 | 0.151 | 4 | -400.712 | -385.647 |
| Sex + total alcohol consumed | 0.076 | 0.096 | 2 | -399.037 | -388.994 |
| Sex + alcohol consumed per kg of body weight | 0.074 | 0.094 | 2 | -398.830 | -388.786 |
| Alcohol consumed per kg of body weight | -0.003 | 0.008 | 1 | -392.519 | -384.987 |
| Total alcohol consumed | 0.010 | 0.021 | 1 | -393.703 | -386.170 |
| AUDIT | -0.010 | 0.001 | 1 | -391.925 | -384.393 |
| Sex + AUDIT | 0.083 | 0.103 | 2 | -399.717 | -389.674 |

^a^Midpoint BAC elimination denotes the time (in minutes) from cessation of drinking to the midpoint of an elimination phase.

Adj R^2^: adjusted coefficient of determination, AIC: Akaike Information Criterion, AUDIT Alcohol Use Disorders Identification Test, BIC: Bayesian Information Criterion, df: degrees of freedom, BMI: body mass index, NCS: Natural Cubic Spline, V_d_: volume of distribution, TBW: total body water based on the Watson et al. equation with height, weight, and age (Maskell et al., 2019).

Reference

Maskell PD, Jones AW, Savage A, Scott-Ham M (2019) Evidence based survey of the distribution volume of ethanol: comparison of empirically determined values with anthropometric measures. Forensic Sci Int 294:124-131. https://doi.org/10.1016/j.forsciint.2018.10.033
